# Designing a TiO_2_-MoO_3_-BMIMBr nanocomposite by a solvohydrothermal method using an ionic liquid aqueous mixture: an ultra high sensitive acetaminophen sensor†

**DOI:** 10.1039/d3ra02611f

**Published:** 2023-07-14

**Authors:** Anita K. Tawade, Ajay P. Khairnar, Jayashri V. Kamble, Akash R. Kadam, Kiran Kumar K. Sharma, Anil A. Powar, Vijay S. Patil, Manohar R. Patil, Sawanta S. Mali, Chang Kook Hong, Shivaji N. Tayade

**Affiliations:** a School of Nanoscience and Biotechnology, Shivaji University Kolhapur 416004 Maharashtra India; b R. F. N. S. Senior Science College Akkalkuwa 425415 Maharashtra India patilvs55@gmail.com; c Department of Chemistry, Shivaji University Kolhapur 416004 Maharashtra India snt_chem@unishivaji.ac.in; d Department of Chemistry, Walchand College of Engineering Sangli 416415 Maharashtra India; e Nanochemistry Research Laboratory, G. T. Patil Collage Nandurbar 425412 Maharashtra India; f Department of Advanced Chemical Engineering, Chonnam National University Gwangju 61186 South Korea

## Abstract

This study shows a simplistic, efficient procedure to synthesize TiO_2_-MoO_3_-BMIMBr nanocomposites. Powder X-ray diffraction, scanning electron microscopy, energy-dispersive X-ray spectroscopy, and X-ray photoelectron spectroscopy have all been used to completely analyse the materials. The detection of acetaminophen (AC) has been examined at a modified glassy carbon electrode with TiO_2_-MoO_3_-BMIMBr nanocomposites. Moreover, the electrochemical behavior of the nanocomposite modified electrode has been studied by cyclic voltammetry (CV), differential pulse voltammetry (DPV), chronoamperometry and electrochemical impedance spectroscopy (EIS). The linear response of AC was observed in the range 8.26–124.03 nM. The sensitivity and detection limits (S/N = 3) were found to be 1.16 μA L mol^−1^ cm^−2^ and 11.54 nM by CV and 24 μA L mol^−1^ cm^−2^ and 8.16 nM by DPV respectively.

## Abstract 2

Schematic representation of synthesis and applications of TiO_2_-MoO_3_-BMIMBr nanocomosite for detection of acetaminophen.

## Graphical abstract

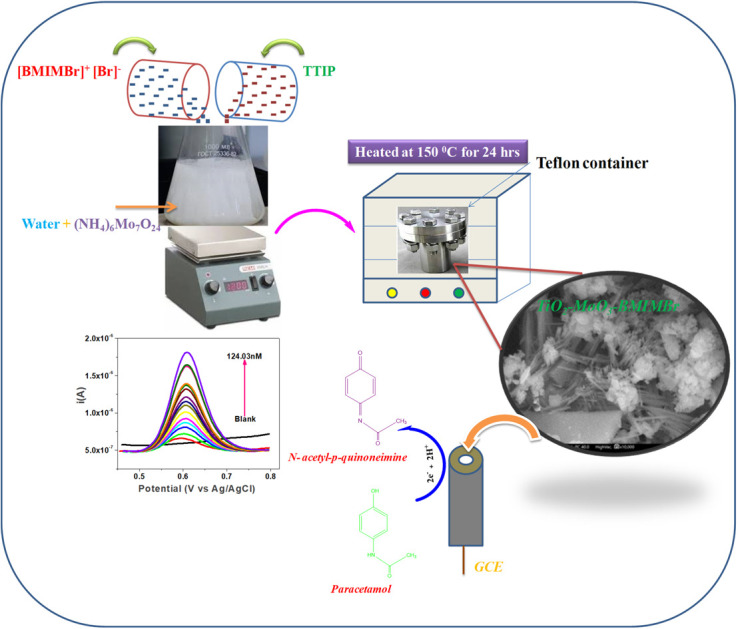

## 1. Introduction

The pharmaceutical industry grows to keep up with the increase in the world's population; the pharmaceutical industry's global market hit $1.25 trillion in 2019; by 2023, it is expected to reach $1.5 trillion. Pharmacy sales revenue increased by 207.9% from 2005 to 2019.^1^ Consequently, two significant problems have appeared. Ambient water sources that contain pharmaceutical pollutants must be tracked and monitored for the preservation of ecological and human health because (i) many pharmaceuticals are distorted, necessitating the need for portable, affordable, and accurate sensors for a variety of applications, including the tracking of drug overdoses or for biomedical monitoring applications, (ii) pharmaceutical pollutant in ambient water sources.

From February 2018 through February 2019, the National Center for Health Statistics recorded 69 029 deaths attributed to non-steroidal anti-inflammatory drug overdoses.^2^ This is an increase of 5.5% from 2017 to 2018. These indicate an increase in antidepressant overdose deaths in the US in 2017, with over 5000 deaths compared to 3889 in 2010.^3^ Between 2005 and 2018, the death rate from antidepressant overdose climbed by an average of 6% a year. The cumulative content of the 37 rivers tested nationwide for antibiotics by the Japanese health authorities was as high as 626 ng L^−1^.^2^ The WHO recommends an adult daily intake of 1 g every 4–6 hours and not more than 4 g acetaminophen (AC) in a 24 hour period. For instance, the hydrolytic breakdown of paracetamol results in the production of 4-aminophenol, and it has teratogenic and nephrotoxic consequences. The ambient aquatic systems of 71 countries contained a total of 203 medicines with substantial quantities, the majority of which were antibiotics, analgesics, pain relievers, anti-fungal and anti-inflammatory drugs according to a report by Germany's Ministry for the environment.

The analgesic and antipyretic medication paracetamol (4-acetaminophen or *N*-acetyl-*para*-aminophenol) (POM) is used to treat pain, neuralgia, arthritis, and menstrual cramps in both adults and children.^4^ Limited use of AC does not exhibit any harmful side effects, however, excessive use or overdose and chronic use of AC will cause kidney, liver damage and even coma or death in some cases.^5^ Thus, the monitoring of POM in human serum, sweat, and water bodies are important for both human health and controlling environmental poisoning.^5^ Numerous techniques such as high performance liquid chromatography,^6^ UV spectrophotometry,^7^ chemiluminescence^8^ and titrimetry,^9^ electrophorosis,^10^ LC-MS^11^ GC-MS^12^ and flow injection^13^ are available for the detection and determination of POM. Unfortunately, these techniques have several drawbacks and restrictions, such as being expensive and time-consuming. Electrochemical methods have been extensively researched for the determination of POM due to its advantages of being straightforward, affordable, sensitive, and selective analytical techniques with quick analysis and precise results.^14^

Due to its biocompatibility and advantages in terms of cost, TiO_2_ is particularly preferred for the creation of biosensors and electrochemical sensors.^15^ Due to their partially filled orbitals, transition metal oxides (TMO) have unique qualities that make them stand out, like being environmentally friendly, simpler to manufacture, having a large specific surface area, and broad energy band gap, being biocompatible, and more.^16^ MoO_3_ are widely used in catalysts, photoelectrochemical devices, gas sensors, electrochemical capacitors, and other fields with their excellent physical and chemical properties.^17^ However, the performance of TMO materials alone is not always ideal. However, due to their inherent disadvantages, such as poor electron conductivity, low stability in acidic media, and weak adsorbate adsorption and activation capacities, there are some key challenges in the practical applications of TMOs. In order to improve their physical and chemical properties, several innovative strategies have been developed. Xu^18^ used chemical deposition to grow uniform porous NiCo_2_O_4_ nanosheets on the surface of α-MoO_3_ nanorods, and achieved a detection limit as low as 50 ppb. The heterostructure nanomaterials formed have excellent gas-sensitive sensing performance for ethanol. The Ag–P NPs synthesized by simply refluxing are deposited on the screen-printed carbon electrode (SPCE) to detects POM in the linear concentration of 0.1–1900 μM with the lowest detection limit of 0.39 nM (S/N = 3) with the superior sensitivity of 2244.4 μA μM^−1^ cm^−2^.^19^

The numerous methods are developed for AC sensing *viz* titrimetric, spectrophotometric, and chemiluminescence. These methods require an extraction step before detection, whilst liquid chromatography is a laborious procedure, making these methods unsuitable for regular inspection. Fundamentally non-specific are conductometric approaches. However, there are a few pragmatic aspects that make conductometric approaches distinct, such as their cost-effectiveness, simplicity (because no reference anodes are needed), and insensitivity to light. Chromatographic methods including HPLC, GC-MS, and LC-MS/MS as well as colorimetric techniques are being used extensively in hospitals and laboratories for the analysis of pharmaceutical substances.^20^ However, these methods frequently have limitations when it comes to mobility. Our main objective is to synthesize morphologically controlled electrode as a low-cost, highly sensitive, and highly stable modifier utilizing a simple synthesis process in order to satisfy the parameters for the ideal chemically modified electrode. Van der Waals force, extended hydrogen bonds, and electrostatic interaction, for instance, all exist in ionic liquid. These properties of ILs that provide a specific interaction with metal nanoparticles allow for the creation of inorganic materials with a range of morphologies.^21,22^ The AgNPs are progressively increase in the soil and water reservoir. Inhalation of AgNPs in human tissue cells causes increase cytotoxicity and the concentration of reactive oxygen species (ROS). It is difficult to maintain the active state AgO in oxygen atmosphere therefore AgNPs possibly loose response for sensing of AP compared to the transition metal oxides. To the best of the authors' knowledge, ionic liquid-based TiO_2_-MoO_3_ has not yet been used for POM electrochemical sensing.

Among various preparation methods for TiO_2_-MoO_3_-1-butyl-3-methylimidazolium bromide (BMIMBr), the simple one-pot hydrothermal preparation approach can avoid introduction of impurities and toxic reducing agents, but the rate of nucleation, growth and aggregation of metal nanoparticles are out of control. Opportunely, ionic liquid (IL) has low interface tension and can enhance nucleation rate.^23^ Ultrafine metal NPs can be generated, which only undergo weak Ostwald ripening.^24^ ILs plays crucial role for the establishment of the protective layer at the particle surface and the subsequent electrosteric, solvation, and viscous stabilization of developing particles, their polarity and affinity towards particles and precursors, transport, and surface qualities appear to be particularly important.^25^ Furthermore, ILs is favorable to the formation of a soft sensing-interface, benefiting the recognition/rebinding of template molecule.

In this work, we have developed a simple electrochemical sensing platform based on solvohydrothermally prepared TiO_2_-MoO_3_-BMIMBr nanocomposite in the mixture of ILs and water for the sensitive detection of POM in pharmaceutical formulations and biological samples. The synthesized nanomaterials were characterized in detail using several advanced characterization techniques. The electrochemical performance of TiO_2_-MoO_3_-BMIMBr modified glassy carbon electrode (GCE) was evaluated by CV, DPV and chronoamperometry techniques. The modified GCE exhibits appreciable electrocatalytic activity towards POM oxidation and the modified electrode displayed a wide linearity, good sensitivity, selectivity, and high stability for the detection of POM. The TiO_2_-MoO_3_-BMIMBr/GCE based sensor has also been successfully used to measure POM in human urine samples and pills. Hence, the current study offers a new door for the electrochemical method of POM estimate employing a basic electrochemical sensing platform.

## 2. Experimental

### 2.1 Chemical reagent

Methyl imidazole (Sigma Aldrich, 99%), 1-bromo butane (C_4_H_9_Br), (Sigma Aldrich 99%), toluene (C_6_H_5_CH_3_) pub chem, titanium isopropoxide (Ti[OCH(CH_3_)_2_]_4_) Sigma Aldrich, ammonium heptamolybdate ((NH_4_)_2_MoO_4_) 99.98% trace metals basis, nitric acid (60–79%), Thomos baker. Dolo (paracetamol) 650 mg. Acetic Acid (99%) and sodium acetate (99%) (Thomos baker). All additional chemicals were of analytical quality, and double-distilled water was used to make all solutions.

### 2.2 Instrumentation

With a rotating anode X-ray generator operating at Cu–K monochromatic radiation (=1.5418), a Bruker D8 Advance X-ray diffractometer (XRD) was used to evaluate the phase structures and crystalline size of the samples as-prepared. Thermo Scientific's XPS (VG Multilab 2000-Thermo Scientific, USA, K-Alpha), which can withstand high photonic energies of 0.1 to 3 keV, was used to gather the X-ray photoelectron spectra (XPS). The FT-IR-6600 spectrometer, manufactured by the Bruker business, was used to record FT-IR spectra. The spectrum was obtained using KBr, and the data was stored on an IFS 66V/S. Particle size and charge on material studied by using Litesizer 500 particle analyzer made by Anton Parr. All the electrochemical experiments were performed with (Autolab Metrohm PGSTAT 302N). All electrochemical experiments are performed with three eletrode systems glassy carbon electrodes (GCE), C110 of 3 mm diameter GCE used as working electrode or modified electrode, a counter electrode (Pt) and a reference electrode Ag/AgCl (in saturated KCl solution). All potentials in this paper were measured and reported *versus* Ag/AgCl. It is worth mentioning that in this study, and all experiments were carried out at laboratory temperature.

### 2.3 Preparation of modified electrode

The modification, GCE (model C110, 3 mm diameter) was polished before each experiment with 0.05 μm alumina power, rinsed thoroughly with double distilled water between each polishing step, and then sonicated successively in 1 : 1 nitric acid, absolute alcohol, double distilled water. The cleaned electrode was air dried. To prepare the modified electrodes, 2 mg of the active materials were dispersed into 2 ml ethanol to give homogeneous suspension upon bath sonication. 10 μL of the suspension was dropped (*n* = 3) onto GCE and the electrode was then dried at room temperature (RT). The modified electrode use further for electrochemical performance with paracetamol.

### 2.4 Synthesis of ionic liquids [BMIM]^+^[Br]^−^

1-Butyl-3-methylimidazole bromide was prepared by a dropwise addition of 1-bromobutane (2.4 mol) to *n*-methylimidazole (1.85 mol) and toluene (0.13 mol) in round bottom flask at temperature 8 °C. The reaction was allowed to proceed at RT for 72 h. Afterwards, ionic liquid was extracted by using diethyl ether (25 mL × 2). Extracted ionic liquid was dried at temperature 60 °C for 24 h. Then ionic liquid was preserved in sealed container for further use.

### 2.5 Synthesis of TiO_2_-BMIMBr

6.375 g TTIP, 6 ml BMIMBr ILs mixed in 25 ml of distilled water (DW). The mixture was stirred well to get a white precipitate. The obtained precipitate was dissolved using concentrated HNO_3_. The obtained clear solution was loaded into autoclave heated at 160 °C for 24 h. After cooling to RT, the product was isolated and purified by centrifugation and sequential washing with water and ethanol 4–5 times. The product was dried at 80 °C for 3 h and then annealed at 600 °C.

### 2.6 Synthesis of MoO_3_-BMIMBr

1.8 g ammoniumheptamolybdtae, 6 ml BMIMBr ILs mixed in 25 ml of DW. The mixture was stirred well to get a white precipitate. The obtained precipitate was dissolved using concentrated HNO_3_. The obtained clear solution was loaded into autoclave heated at 160 °C for 24 h. After cooling to RT, the product was isolated and purified by centrifugation and sequential washing with water and ethanol 4–5 times. The product was dried at 80 °C for 3 h and then annealed at 600 °C.

### 2.7 Synthesis of TiO_2_-MoO_3_-BMIMBr nanocomposite

Ionic liquid [BMIM]^+^[Br]^−^ and DW each 12.5 ml were mixed into 100 ml round bottom flask and stirred at 150 rpm. Titanium isopropoxide (TTIP) (3.9085 ml) and ammonium heptamolybdate (3.489 g) were added into the mixture. The reaction leads to a white precipitate of the desired composition. The required amount of nitric acid was added to dissolve the white precipitate. The obtained broth poured into a Teflon-coated stainless steel autoclave hydrothermal cell. The autoclave was kept in furnace for 24 h at 150 °C. The mixture was collected and centrifuged using isopropanol. The semi-solid product was transferred to a Petri dish and dried for three hours in a hot air oven at 80 °C and annealed at 600 °C.

## 3. Results and discussion

**Fig. 1:**
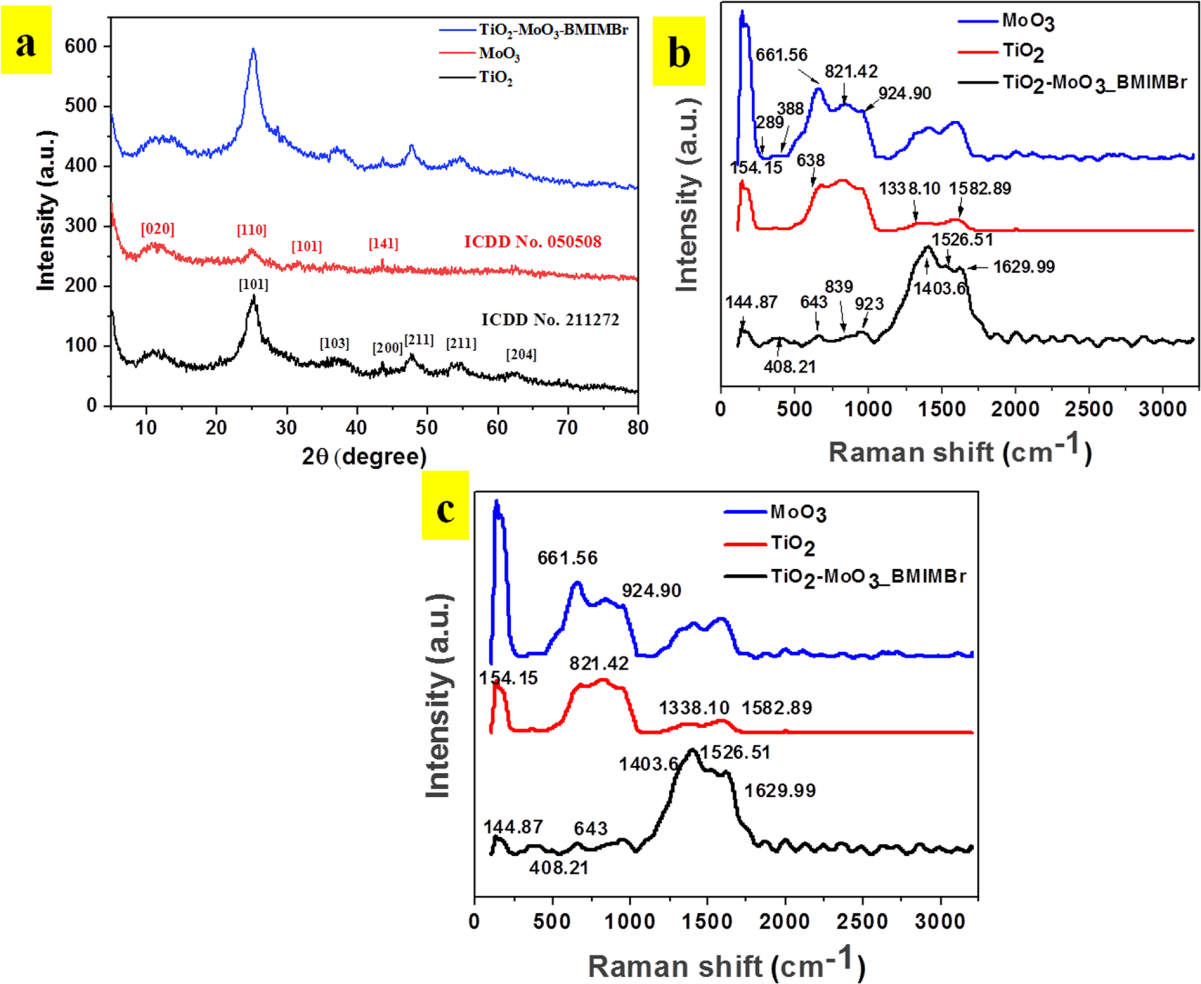
(a) XRD, (b) Raman and (c) FT-IR pattern of synthesized TiO_2_-MoO_3_-BMIMBr nanocomposite.

The X-ray diffraction patterns of TiO_2_, MoO_3,_ and TiO_2_-MoO_3_-BMIMBr nanocomposite as shown in Fig. 1, The relevance of the nanocomposite with the standard ICDD data cards of TiO_2_ and MoO_3_ is attributed to the crystalline nature of the synthesized nanocomposite. The synthesized MoO_3_ crystallizes in a hexagonal structure (ICDD card No. 050508). Crystalline planes such as (020), (110), (101), (141), were observed for the MoO_3_ sample. For the reference TiO_2_ sample, the recorded diffraction peaks assigned to the rutile TiO_2_ crystalline structure and the respective crystalline planes are (101), (103), (200), (211) (204). From the diffraction patterns, the TiO_2_-MoO_3_-BMIMBr nanocomposite, strong and high intensity peaks corresponding to (020), (101), (103), (200) (211) (204) characteristic for both rutile TiO_2_ and α-MoO_3_ planes crystal structure and phases. The XRD analysis confirms the rutile TiO_2_ and alpha morphology of MoO_3_ in the nanocomposite. Using the Scherrer formula and the full width half maximum (FWHM) of the high intensity diffraction peak, the grain size was determined eqn (1)1
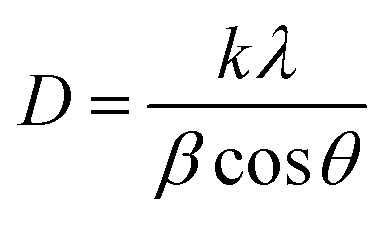
where, *k* is a numerical constant (0.94), *λ* is the X-ray wavelength (1.54056 Å), *θ* is the angle of the corresponding peak and *β* is the FWHM (full width at half maxima) of the corresponding peak. The average crystalline sizes (*D*) of the pure MoO_3_, TiO_2_, and TiO_2_-MoO_3_-BMIMBr are 3.53, 0.249 and 3.32 nm, respectively. Also, due to the alkyl chain of the imidazolium cation, a hump-like broad peak at 30–34° appeared.

Raman spectra of pristine TiO_2_, MoO_3_ and TiO_2_-MoO_3_-BMIMBr nanocomposite are shown in Fig. 1b. The Peaks at 145, 379, 521, 671 cm^−1^ are the characteristic peaks of TiO_2_^.^ Also the characteristic peaks of MoO_3_ E_g_(1), B_1g_(1), A_1g_ or B_1g_(2), and E_g_(3) modes are found in TiO_2_-MoO_3_-BMIMBr nanocomposite respectively. This confirms the characteristic signatures of TiO_2_ and MoO_3_ in the nanocomposite. The relative intensities of the peaks in Fig. 1b at 643–652 cm^−1^ differ depending on the halogen anion. These peaks are assigned to the vibrational modes of the imidazolium ring; the 643 cm^−1^ band corresponds to the trans conformation of the C7–C8 bond of the *n*-butyl group and the 652 cm^−1^ peak corresponds to the gauche conformation.^26^ The detailed molecular vibration and rotation information of α-MoO_3_ and h-MoO_3_ are evidenced by Raman spectrum (Fig. 1b). For α-MoO_3_, the characteristic peak at 388.94 cm^−1^ is attributed to –O–Mo–O– scissoring, and 289 cm^−1^ to –O=Mo=O– wagging. Moreover, the peaks at 661.56 cm^−1^ (B_2g_, B_3g_ asymmetric stretching of –MO–O–MO– are due to stretching; the 821.42 cm^−1^ and 994.90 cm^−1^ peaks are due to the stretching of terminal –Mo=O– bonds^27^ respectively. The intense rutile TiO_2_ Raman vibrations at 154.14 cm^−1^ (E_g_), 201 cm^−1^ (E_g_), 408.21 cm^−1^ (B_1g_) and 643 cm^−1^ (E_g_) corresponding to the E_g_ and A_1g_ vibration modes.^28^ The peaks situated at 276.18 cm^−1^ (B_2g_, B_3g_), 361.11 cm^−1^, 849.96 cm^−1^ symmetric stretching of terminal molybdenum to oxygen double bond and 952.73 cm^−1^ –MO–O–MO– linkage are the Raman characteristics of α-MoO_3_ (Table 1).^29^

**Table 1:** Assigned peaks of the functional groups using Raman spectroscopy

Wavenumber (cm^−1^)	Functional group
144.87	E_g_(1) mode
379	B_1g_(1) mode
521	A_1g_ or B_1g_(2) modes
671	E_g_(3) mode
643	*Trans* conformation of the C7–C8
652	*Gauche* conformation
388.94	–O–Mo–O– scissoring
289	–O=Mo=O– wagging
661.56	B_2g_, B_3g_ asymmetric stretching of (–MO–O–MO–)
821.42 and 994.90	–Mo=O–
154.14	E_g_ mode
201	E_g_ mode
408.21	(B_1g_)
643	E_g_ and A_1g_ vibration modes

FTIR spectrum was performed to investigate chemical bonding states between molybdenum, titanium and oxygen atoms as shown in Fig. 1c. The intense peak at 557 cm^−1^ is assigned to the –Ti–O– stretching band which is the characteristic peak of TiO_2_.^30^ The spectrum in Fig. 1c shows a number of bands that are associated with surface-bound water, including the –O–H mode at 3147 cm^−1^ and deformation mode at 1615 cm^−1^. The –Mo–OH vibrational mode appears at 1406 cm^−1^, and was associated to the interaction of the Mo with some water in the surface. Bands arising from the Mo vibrations appear at 918 cm^−1^, being assigned to the vibrations of –Mo=O–.

The basic information in FTIR of the TiO_2_-MoO_3_-BMIMBr nanocomposites shows that at lower wavenumber of MoO_3_, the characteristic –Mo=O– asymmetric stretching band at 918 cm^−1^.^31^ Moreover, these bands affirm the formation of monolayer over TiO_2_ surface at lower MoO_3_ loadings, which is in correlation with the FTIR results.^32^ Before to the completion of monolayer coverage, the more aggregated polymolybdate structures cause a minor blue-shift in the 918 cm^−1^ band at MoO_3_ loadings.^33^ Therefore, FTIR and Raman analyses emphasize the formation of surface interaction of molybdate species within the monolayer at lower loadings of MoO_3_ in TiO_2_ with BMIMBr ionic liquid (Table 2).

**Table 2:** Characteristics FTIR absorption frequencies

Functional group	Absorption location (cm^−1^)	Absorption intensity
–Ti–O–	557	Medium
–Mo=O– (asymmetric stretching)	918	Weak
–Mo–OH	1406	Medium
–O–H (deformation mode)	1615	Strong
–O–H mode	3147	Medium, broad

### 3.1 Dynamic light scattering (DLS) and zeta potential analysis

**Fig. 2:**
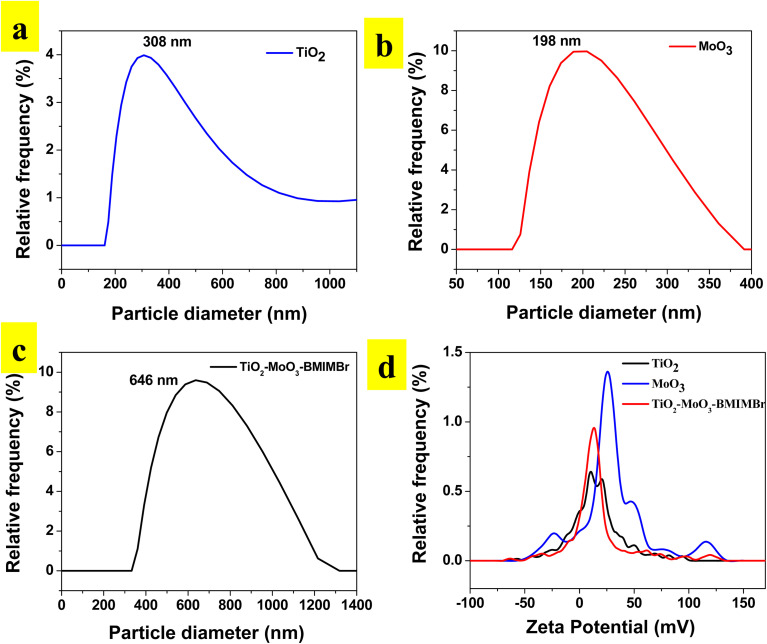
Particle distribution of (a) TiO_2_, (b) MoO_3_, (c)TiO_2_-MoO_3_-BMIMBr nanocomposites and (d) zeta potential plot overlay.

To analyze the particle size distribution of synthesized TiO_2_-MoO_3_-BMIMBr nanocomposite were subjected to dynamic light scattering (DLS) analysis. Here material was dispersed completely in ethanol using ultra-sonicator for DLS analysis. Results obtained as shown in Fig. 2a, reveals the monodisperse nature of particles with an average particle size was around 308 nm for TiO_2_, 198 nm for MoO_3_ and TiO_2_-MoO_3_-BMIMBr shows 646 nm. The enhanced particle size in composite observed due to ILs. As shown in Fig. 2b, zeta potential was employed to evaluate the stability of nanoparticles. The TiO_2_, MoO_3_ and TiO_2_-MoO_3_-BMIMBr zeta potential value was discovered to be 1.9 mV, 26.6 mV and 11.2 mV. The significance of zeta potential (ZP) is that its value can be related to the stability of the TiO_2_-MoO_3_-BMIMBr nanocomposite and pristine nanomaterial. From the ZP measurements, the MoO_3_ is found highly stable compared to TiO_2_ and nanocomposite. The decrease in the stability in case of nanocomposite possibly due to improper orientation of bulky cation of ionic liquid in the stern layer.

### 3.2 Scanning electron microscopy

**Fig. 3:**
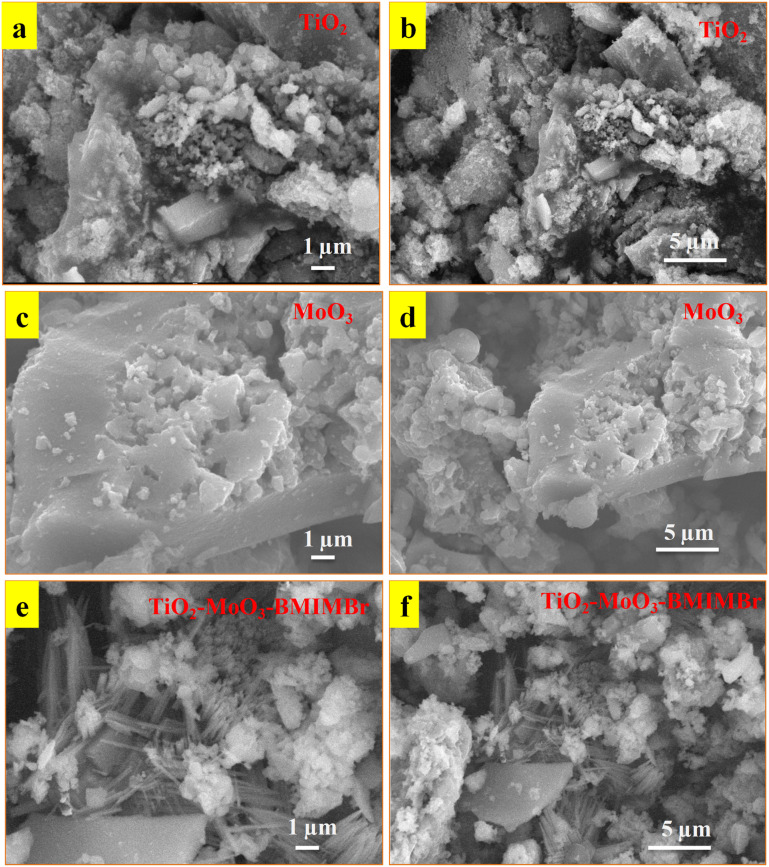
SEM images of synthesized TiO_2_ (a and b), MoO_3_ (c and d) and TiO_2_-MoO_3_-BMIMBr nanocomposite (e and f).

The morphology of synthesized nanomaterial determined by using SEM analysis. Obtained results as show in Fig. 3a and b reveals that the synthesized TiO_2_ nanoparticles and aggregated into an irregular structure at 1 μm resolutions. SEM images show the compactly arranged and uniformly deposited TiO_2_ nanoparticles over the substrate. The MoO_3_ shows sheet like morphology at 1 μm and 5 μm resolution. The SEM images of TiO_2_-MoO_3_-BMIMBr nanocomposites are demonstrated in Fig. 3e and f. These structures exhibited similar morphological characteristics with TiO_2_/MoO_3_. These structures exhibited similar morphological characteristics with TiO_2_/MoO_3_. The TiO_2_ nanoparticles and rod like morphlogy of ILs having thickness is at nanoscale adsorbed on the surface of sheet of MoO_3_ exhibit higher surface area.

**Fig. 4:**
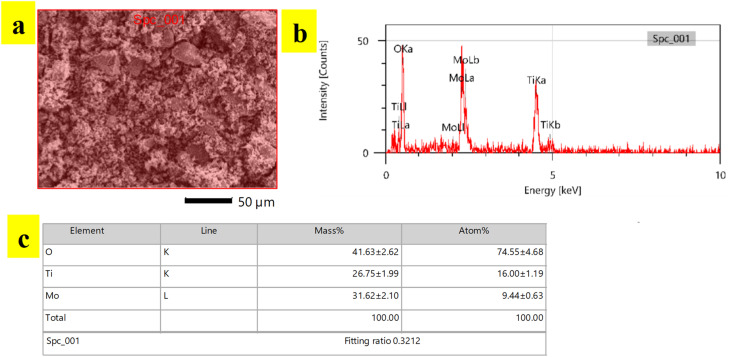
EDS of TiO_2_-MoO_3_-BMIMBr nanocomposites.

Compositional analysis of synthesized TiO_2_-MoO_3_-BMIMBr nanocomposite was done by EDS analysis. EDS spectra recorded during the SEM imaging technique and are shown in Fig. 4. The peaks around 2 to 3 keV are from the MO LI, MO Lb and MO La lines, and peaks observed with less counts are observed at 0.452 keV and 0.458 keV are due to Ti La and Ti LI line. The peaks around 0.5 keV are from the O Ka lines. Peaks observed at 4.5 keV and 4.9 keV are due to Ti Ka and Ti Kb line. The reason why electrons from the L and M shells fall back to form discrete X-ray quanta from L to K yields Ti Ka1 at 4.5 keV and from L to K gives Ka2 is because the incident electron will remove an electron from the Ti K shell. The inset picture shows the EDS of TiO_2_-MoO_3_-BMIMBr nanocomposite. As a result of the incident electron removing one electron from the Ti K shell, discrete X-ray quanta are produced when electrons from the L and M shells fall back to the Ti K shell (Ti Ka1 at 4.5 keV) and the Ti K shell (Ka2). It is observed that the nanoparticles contain molybdenum, titanium and oxygen elements predominantly. This confirms the presence of TiO_2_, MoO_3_ particles.

**Fig. 5:**
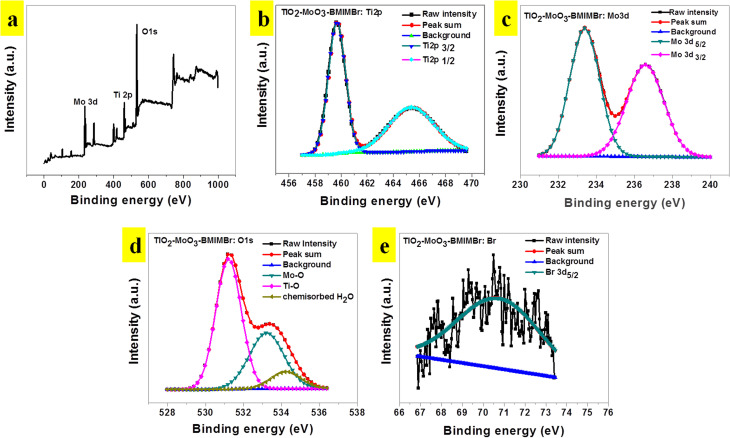
(a) XPS survey spectra, (b) Ti 2p (c) Mo 3d and (d) O 1s signals and (e) Br 3d of TiO_2_-MoO_3_-BMIMBr nanocomposite.

Fig. 5a shows the XPS survey spectrum of the representative TiO_2_-MoO_3_-BMIMBr nanocomposite, showing the existence of the elemental compositions of Mo 3d, Ti 2p, O 1s and Br in the TiO_2_-MoO_3_-BMIMBr while the absence of other peaks in the nanocomposite indicates that no impurities are present in the TiO_2_-MoO_3_-BMIMBr nanocomposite. The high resolution core level XPS spectrum for Ti 2p is illustrated in the Fig. 5b. It shows the appearances of the two distinct characteristics peaks at 459.57 eV and 465.48 eV corresponding to the Ti 2p_3/2_ Ti 2p_1/2_, respectively. However, it shows the slight shifting of the characteristics peaks to the higher binding energy (233.41 eV and 236.60 eV) values to that of bare Mo 3d_5/2_ and Mo 3d_3/2_ nanoparticles as shown Fig. 5c. Fig. 5d shows the high resolution core level O 1s XPS spectrum of the representative TiO_2_-MoO_3_-BMIMBr three distinct peaks it shows the strong peak at 531.25 eV assigned due to the characteristic binding energies for lattice oxygen of TiO_2_ and again it deconvoluted into two peaks at 533.26 eV and 534.17 eV corresponding to –Mo–O– binding and chemisorbed water. The Br 3d_5/2_ XPS spectrum comprises of Br 3d_5/2_.

### 3.3 Electrochemical properties measurements

**Fig. 6:**
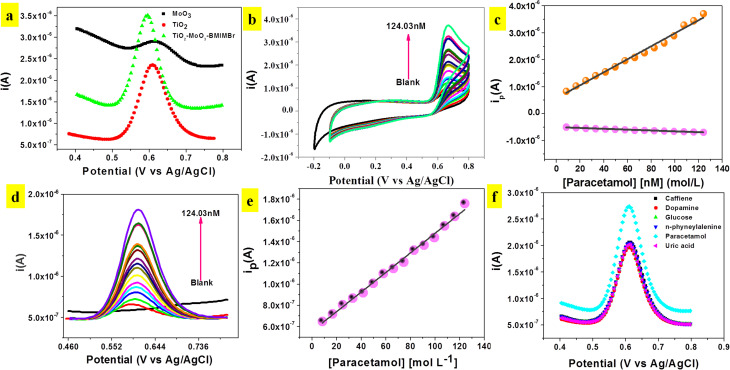
(a) DPV responses at the MoO_3_, TiO_2_ and TiO_2_-MoO_3_ nanocomposite of 33.07 mM POM in 0.1 M ABS, pH 2 at a scan rate of 50 mV s^−1^ (b) CV curves recorded at different POM concentration (8.26–124.03 nM) at the TiO_2_-MoO_3_-BMIMBr nanocomposite. (c) Corresponding redox peak *vs.* concentration calibration plot (d) DPV responses obtained at the nanocomposite for the successive addition of POM (8.26 nM–124.03 nM) in 0.1 M ABS, pH 2.0. DPV conditions: *E*_step_ −0.005 V, amplitude 0.025, modulation time 0.05 s. (e) Corresponding calibration plot. (f) Influence of different interfering compounds on the *i*_pa_ detected at TiO_2_-MoO_3_-BMIMBr/GCE.

The phenolic hydroxyl group in paracetamol (POM) makes it electrochemically active and susceptible to oxidation. Hence, the electro catalytic behaviour of the modified TiO_2_-MoO_3_-BMIMBr electrode was examined. Using the Differential Pulse Voltammetry (DPV) technique with a scan rate of 50 mV s^−1^, the electrochemical behaviours of various modified glassy carbon electrodes (MoO_3_, TiO_2_, TiO_2_-MoO_3_-BMIMBr/GCE) in the presence of 0.1 M ABS pH 2 were investigated. Compared with the other modified electrode TiO_2_-MoO_3_-BMIMBr/GCE, the peak current signals at nanocomposite are much larger, indicating the strong electro catalytic activity for POM (82.6 nM) as shown in Fig. 6a. Fig. 6b shows the cyclic voltammograms of nanocomposite in 0.1 M of ABS with different concentration of POM from 8.26 nM–124.03 nM. The modified nanocomposite electrode successfully participates in the reversible electrochemical redox process of POM, thereby indicating a very fast and direct electron transfer of POM at the modified electrode (*E*_pa_ = 0.66 V, *E*_pc_ = 0.13 V). The oxidation peak current progressively rises with rising POM concentration, as can be seen. Fig. 6 displays the related calibration curve (peak current *vs.* POM concentration) (c). From 8.26 nM to 124.03 nM, the calibration curve shows linearity throughout the whole dose range. With a correlation coefficient *R*^2^ of 0.99, the sensitivity and limit of detection were 1.1598 A L mol^−1^ cm^−2^ and 11.54 nM, respectively.

Fig. 6d illustrates DPV of TiO_2_-MoO_3_-BMIMBr nanocomposite for various concentration of POM from 8.26 nM–124.03 nM. From DPV POM oxidation peak observed at 0.60 V. As the concentration of POM increases, the anodic peak current also increases linearly this confirms the oxidation of POM is diffusion controlled. A plot of current density against concentration of POM is shown in Fig. 6e. The sensitivity and linear range were 24 μA L mol^−1^ cm^−2^. The limit of detection (LOD) was calculated to be 8.16 nM by using the formula 3*σ*/*S* (*σ* is the standard deviation of blank and *S* is the slope of calibration curve). Similarly, the corresponding limit of quantification (LOQ = 10*σ*/*S*) was found to be 27.21 nM.

In Fig. 6f biological samples there may be some interfering species which can be oxidized simultaneously along with POM at the electrode surface and may affect the detection tendency. Therefore, it is necessary to study the selectivity of TiO_2_-MoO_3_-BMIMBr nanocomposite modified GCE towards the detection of POM. Prepared sample solutions containing 100 nM POM with possible biological interfering compound shows the comparison of DPV with caffeine, dopamine, glucose, uric acid, *n*-phyneylalenine were measured to record the peak currents. These interferences have no great response in the detection of POM, suggesting the excellent selectivity of nanocomposite for the determination of POM. The main advantage of the TiO_2_-MoO_3_-BMIMBr nanocomposite modified GCE found herewith is its usefulness for the detection of POM in the real samples (urine sample) without the influence of any interferences.

**Fig. 7:**
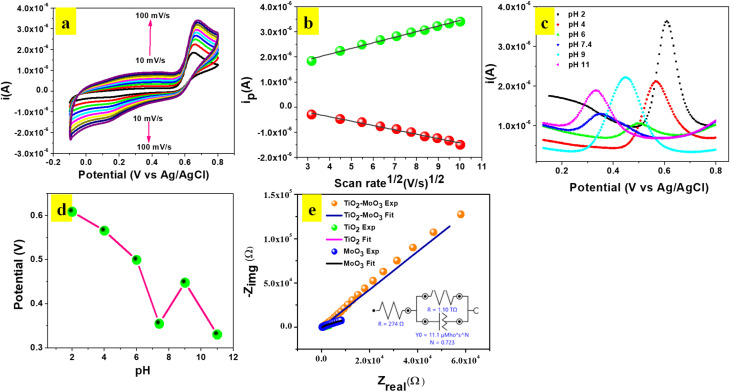
(a) CV responses obtained at the TiO_2_-MoO_3_-BMIMBr nanocomposite sensor for different scan rates (10–100 mV s^−1^) in 0.1 M ABS, pH 2 containing 124.03 nM POM. (b) Linear plot of POM redox peak (*I*_pa_) *vs.* log of scan rate. (c) DPV of effect of pH on modified electrode. (d) The effect of peak potential with respect to pH of the TiO_2_-MoO_3_-BMIMBr/GCE sensor in 0.1 M AB solution. (e) The impedimetric responses towards a 0.1 M ABS with 124.03 nM POM.

CV experiments were carried out to investigate the influence of scan rate at TiO_2_-MoO_3_-BMIMBr/GCE in 0.1 M ABS (pH 2) containing 124 nM POM (Fig. 7a). An evident linear relationship between the square root of the scan rate (*υ*^1/2^) and the oxidation peak current (*I*_p_) in the range of 10–100 mV s^−1^ was obtained. The linear equation was described as: *I*_pa_ = 2.23 × 10^−7^*X* + 1.23 × 10^−6^*υ*^1/2^ (*R*^2^ = 0.99), and *I*_pc_ = −1.74 × 10^−7^*X* + 3.20 × 10^−7^*υ*^1/2^ (*R*^2^ = 0.99), validating the oxidation of POM as a diffusion-controlled process. Meanwhile, there was a positive shift of potential with the increase of scan rate, and the logarithm of scan rate (log *υ*) *vs.* the anodic peak potential (*E*_p_) appeared to have a proportional relationship. This could be due to changes in the electro catalytic activity and kinetic effect of GCE surface on the oxidation of POM especially at scan rates higher than 100 mV s^−1^ the time window for the POM oxidation becomes very narrow, avoiding the facile electron transfer between substrate and catalytic sites.

**Scheme 1:**
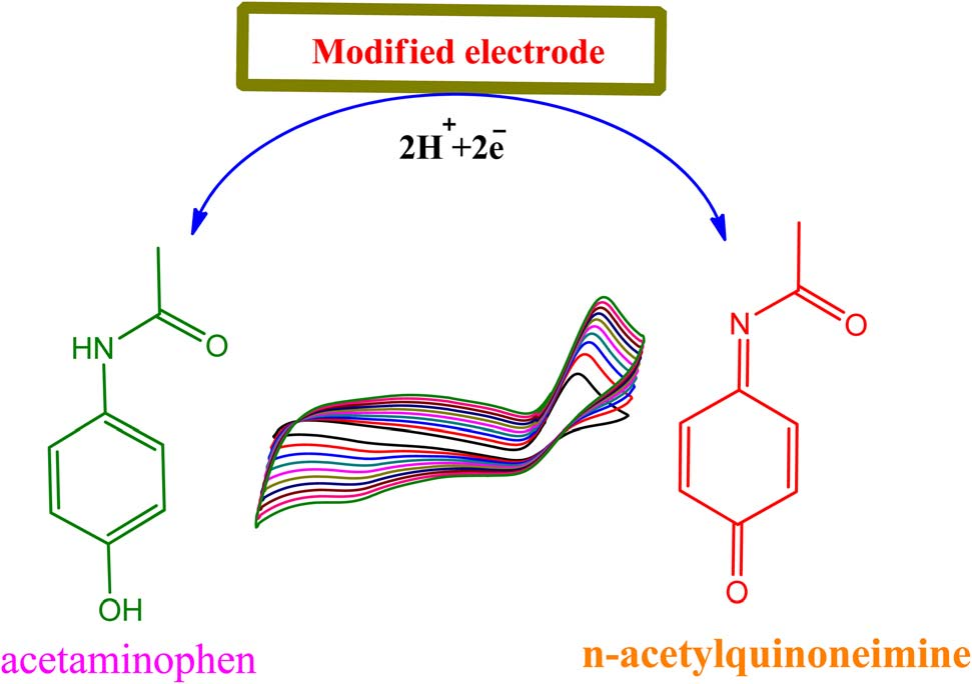
The mechanism of paracetamol oxidation.

The equation for log *υ* and *E*_p_ was: *E*_p_ = 0.0235 log *υ* + 0.627 (*R*^2^ = 0.98). An equation for the irreversible process is described as following:

2 *E*_p_ = 2.303*RT*/2(1 − *α*)*nF* log *n* + *K*

where *α* is the electron transfer coefficient, *R*, *T* and *F* are the gas constant, temperature and Faraday constant *n* is the number of electrons involved in the rate-controlling step, respectively. On the basis of the slope being equal to 2.303*RT*/2(1 − *α*)*nF*, the value of (1 − *α*)*n* is calculated. The electron transfer coefficient *α* is assumed as 0.5 for an irreversible electrode process, then the number of transferred electrons is (*n* = 2) which is consistent with the reported literature.^34^ The electrochemical response of POM depends on pH of solution. Hence, we examined the electro catalytic behavior of TiO_2_-MoO_3_-BMIMBr modified electrode towards POM oxidation at various pH ranges from 2 to 11 in 100 nM POM as shown in Fig. 7c. The oxidation peak decreases with pH and maximum current observed at pH 2. Further increase in pH leads to decrease in oxidation peak current value. Therefore, 0.1 M ABS (pH = 2) was chosen as supporting electrolyte. A small current was detected when the pH of the solution was higher than 2. The broadening of oxidation peaks, *E*_pa_, and decreasing of *i*_pa_ are observed with increasing basicity, at pH higher than 2, suggesting a kinetically less favorable reaction at higher pH. The effect of acetate buffer pH on *E*_pa_ has been investigated in the Fig. 7d. The effect of pH with respect to current (*i*_pa_) as shown in Fig. S1a.† This suggests that the number of protons and electrons transferred in the redox reaction of POM are equal and likely to be two. Thus, the mechanism in Scheme 1 for oxidation of POM is proposed.

The electron transfer kinetics of TiO_2_-MoO_3_-BMIMBr are further explored. Thus, the impedance spectra of TiO_2_, MoO_3_, and nanocomposite modified electrode in 0.1 M AB solution are shown in Fig. 7f. All the impedance spectra are fitted with a detailed fitting data (Table 3). The charge transfers resistances taking place at the solution interface (*R*_ct_) of different modified electrode are 400, 411, 274 Ω, respectively. The *R*_ct_ of nanocomposite is smaller than TiO_2_, MoO_3_, which means that the nanocomposite promotes electron transfer. It is confirmed that TiO_2_-MoO_3_-BMIMBr has the better electron transfer ability for the lowest *R*_ct_.

**Table 3:** EIS spectra *R*_s_ and *R*_ct_ values of synthesized TiO_2_,MoO_3_ and TiO_2_-MoO_3_-BMIMBr nanocomposites

Materials	*R*_s_ (Ω)	*R*_ct_ (TΩ)
TiO_2_	400	3.27 × 10^−8^
MoO_3_	411	4.91 × 10^−8^
TiO_2_-MoO_3_-BMIMBr	274	1.10

### 3.4 Mechanism of interaction between the acetaminophen and modified nanocomposite

The mechanism of paracetamol oxidation is well known in the literature.^35^ On the surface of modified electrode (TiO_2_-MoO_3_-BMIMBr nanocomposite), acetaminophen undergoes electrochemical oxidation in a process that involves the loss of two electrons and two protons producing the product *N*-acetyl-*p*-quinoneimine (Scheme 1) and it dependent on pH value ≥6.0, *N*-acetyl-*p*-quinoneimine stable in deprotonated form and in acidic pH it is rapidly protonated.

### 3.5 Reproducibility, reusability and stability

The assay stability of TiO_2_-MoO_3_-BMIMBr was studied through repeating the determination of 124.03 nM POM by CV. After each determination, the modified electrode used underwent 100 successive CV sweeps between −0.2 and 0.8 V at the rate of 50 mV s^−1^ in the ABS (pH 2) only 20% current drop. After 100 cycles, no more change was observed in the voltammetric profiles of the modified electrode as shown in Fig. S2a.† The fabrication reproducibility of TiO_2_-MoO_3_-BMIMBr was also investigated by chronoamperometry for detecting 124.03 nM paracetamol up to 5000 s were prepared independently by the same procedure, result shows more reproducibility as shown in Fig. S2b.† After that, this modified electrode was used to detect 124.03 nM paracetamol exhibited good reusability with convenience of reuse. Furthermore, when the modified electrode was stored in a RT at it exhibited no obvious decrease in the current response in the more than one month and maintained about 95% of its initial value after one month as shown in (Fig. S2c†). This investigation discloses that the sensor possesses good stability and can be employed for the practical application (Table 4).

**Table 4:** Comparison of the analytical characteristics of various sensing systems for the detection of paracetamol

Modified electrode	Linear range (mM)	Limit of detection (μM)	Sensitivity	Reference
TiO_2_-GR	1.0–100	0.00021	4.04 mA mM	36
MoS_2_TiO_2_/rGO/SPE	0.001–0.125	0.046	0.4425 (μA μM)	37
PEDOT/Au@graphene	0.00015–5.88	0.041	—	38
rGO–PEDOT nanotube	0.001–0.035	0.4	16.85 μA μM^−1^ cm^−2^	39
MoS_2_/TiO_2_ NC modified GCE electrode	0.5–750	0.01		40
Cu_2_O/graphene	0.00002–0.0013	0.0067	—	41
Zn/ZnO/rGO/GCE	0.05–2.0	13	166.5 ± 6 μA mM cm^−2^	42
TiO_2_-MoO_3_-BMIMBr	8.26 nM–124.03 nM	0.0115 (CV), 0.0081 (DPV)	1.1598 μA L mol^−1^ cm^−2^, 24 μA L mol^−1^ cm^−2^	Present work

### 3.6 Real sample analysis

The research is motivated to prepare an electrochemical active material for the practical application. Recovery tests were carried out for assay in human urine samples (voluntary 1 and 2). The samples were obtained from human after 12 h of administration of a tablet containing 500 mg of POM. Prior to analysis, the urine samples were diluted 100 times with ABS. Then the urine sample was added with a known concentration of POM. Using the prepared sensor to detect the concentration of POM in different urine samples before and after spiking, the results obtained were concluded in Table 5. Which indicated that the prepared sensor can be used as an effective and reliable sensing platform for detecting POM in real samples.

**Table 5:** Determination of POM in human urine samples

	Voluntary 1			Voluntary 2		
	Added (nM)	Found (nM)	Recovery (%)	Added (nM)	Found (nM)	Recovery (%)
Unspiked	49.3	50.2	102.0	50.2	49.7	99.1
	99.6	99.0	100.0	82.7	83.9	101.0
	140.0	138.0	98.5	117.0	116.0	99.2
Spiked	41.7	41.0	98.3	41.9	41.0	98.0
	89.2	88.7	99.4	82.7	82.3	99.5
	125	124.0	99.2	125.0	124.0	99.7

## 4. Conclusion

A nanocomposite material TiO_2_-MoO_3_-BMIMBr, spherical in shape was developed and applied for the electrochemical detection of acetaminophen from Dolo 650 mg under neutral conditions. On the other hand, the ILs enhances the nucleation rate and provides the conductive bridges to improve the electron transfer between the rutile TiO_2_ and α-MoO_3_. From the CV measurements, the electrocatalytic ability of the GCE modified with TiO_2_-MoO_3_-BMIMBr nanocomposite shows good linear relationship with the concentration of acetaminophen up to nM range. The quantitative determination of acetaminophen by the TiO_2_-MoO_3_-BMIMBr modified electrode was studied by CV and DPV with a linear range of 8.26–124.03 nm. The sensitivity and detection limits found to be 1.16 μA L mol^−1^ cm^−2^ and 11.54 nM by CV and 24 μA L mol^−1^ cm^−1^ and 8.16 nM by DPV and a detection limit of 0.4 mM were achieved in 0.1 M ABS (pH 2) at an applied potential of 0.60 V. The TiO_2_-MoO_3_-BMIMBr modified electrode also exhibits excellent selectivity for acetaminophen and reproducibility investigated by chronoamperometry for up to 5000 s. The proposed TiO_2_-MoO_3_-BMIMBr based acetaminophen sensor shows high potential for applications for more practical purposes.

## Author contributions

Anita K. Tawade and Ajay P. Khairnar – methodology, experimentation and writing rough draft. Jayashri V. Kamble, Akash R. Kadam, Anil A. Powar: experimentation and analysis. Kiran Kumar K. Sharma, Sawanta S. Mali and Chang Kook Hong: resources. Vijay S. Patil, Manohar R. Patil, Shivaji N. Tayade: supervision, validation, conceptualization and editing original draft.

## Conflicts of interest

All authors declares that there is no financial or any other authorship related conflict of interest amongst the authors.

## Supplementary Material

RA-013-D3RA02611F-s001
