# Supplementary material for: Designing a TiO2-MoO3-BMIMBr nanocomposite by a solvohydrothermal method using an ionic liquid aqueous mixture: an ultra high sensitive acetaminophen sensor
Source: RSC Adv. 2023 Jul 14;13(31):21283–95. doi: 10.1039/d3ra02611f (PMC10345954; doi:10.1039/d3ra02611f)
Supplement: RA-013-D3RA02611F-s001 [file RA-013-D3RA02611F-s001.pdf]

Electronic Supplementary Material (ESI) for RSC Advances.
This journal is © The Royal Society of Chemistry 2023

Designing of TiO_2-MoO_3-BMIMBr Composite By a Solvohydrothermal Method using Ionic Liquid Aqueous Mixture: An Ultra High Sensitive Acetaminophen sensor

Anita K. Tawade[1Ψ], Ajay P. Khairnar[2Ψ], Jayashri V. Kamble[3], Akash R. Kadam[3], Kiran Kumar K. Sharma[1], Anil A. Powar[4], Vijay S. Patil[*2], Manohar R. Patil[5], Sawanta S. Mali[6], Chang Kook Hong[6], Shivaji N. Tayade[*3]

[1]School of Nanoscience and Biotechnology, Shivaji University, Kolhapur, 416004, Maharashtra, India

[2]R.F.N.S Senior Science College Akkalkuwa 425415, Maharashtra, India

[3]Department of Chemistry, Shivaji University, Kolhapur, 416004, Maharashtra, India

[4]Department of Chemistry, Walchand College of Engineering, Sangli, 416415, Maharashtra, India

[5]Nanochemistry Research Laboratory G. T. Patil Collage, Nandurbar, 425412, Maharashtra, India

[6]Department of Advanced Chemical Engineering, Chonnam National University, Gwangju, 61186 South Korea.

Ψ Equal first authors

Corresponding Authors 1: snt_chem@unishivaji.ac.in

Corresponding Authors 2: patilvs55@gmail.com

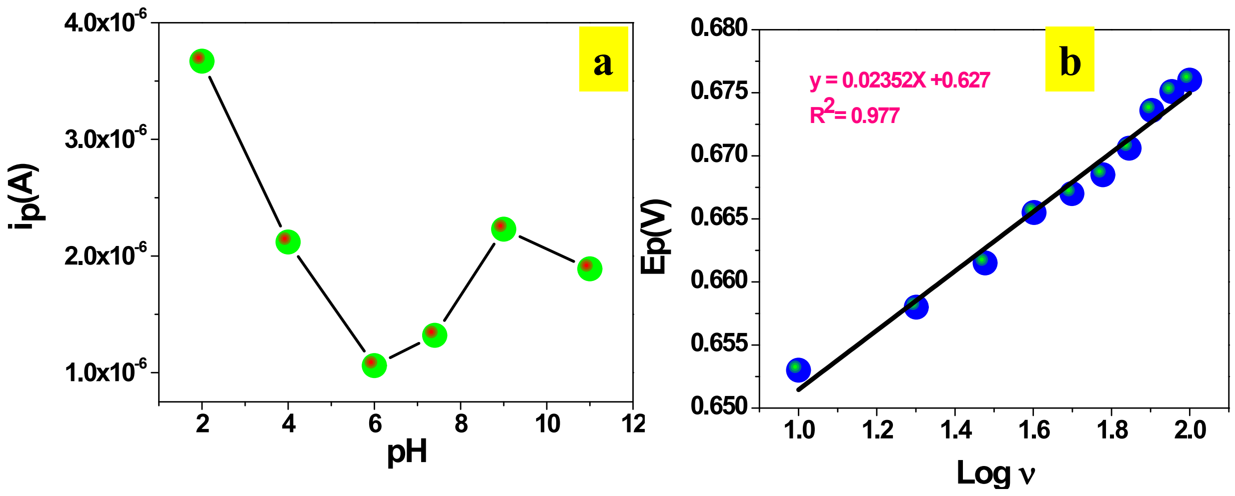

Fig 1S. (a)The effect of peak current with respect to pH of the TiO_2-MoO_3-BMIMBr /GCE sensor in 0.1 M AB solution.(b) Dependence of peak potential(Ep) at different scan rates from 10 to 100 mV s^{-1} log υ.

From Fig. 1S(a) it can be seen that the redox peak current signal decreases with the solution pH ranging from 2to 6, and beyond this pH range, the increase of the redox peak current signal is observed (7.4 to 11). Considering the determination sensitivity, the pH value of 2 in the AB solution was chosen in the following investigation. Interestingly, the anodic peak potentials shift to more negative values with increasing the pH values as shown in Fig. 1S(a). This observation can be explained by the protons taking part in the electrochemical reactions.[1]

To understand the mechanism of mass transport and electron transfer, it is necessary to consider the effect of scan rate. Fig. 1S(b) shows CVs recorded at a different scan rate from 10 to 100 mV s− 1 in 0.1 M PBS, pH 2 containing 124.3 nM of POM. The plot Ep(V) versus the log (scan rate) obtained at the modified electrode(Fig. S(b) showed good linearity, with the following linear regression equation: Ep = 0.023log (v) +0.627.The slope obtained is almost one unity, indicating that the electro oxidation of POM at the TiO2-MoO3-BMIMBr/GCE was completely adsorption-controlled process. Therefore, the current might be proportional to the scan rate according to equation (1)

To determine number of transferred of electrons in POM:

$$Ep = 2.303RT/2(1- \alpha)nF \log \upsilon + K \text{...........eq(1)}$$

$$Slope = \frac{2.303\, RT}{2(1 - \propto)nF}$$

$$n = \frac{2.303 \times 8.314 \times 298}{2 \times 0.5 \times 0.023 \times 96500}$$

$$n = 2.6$$

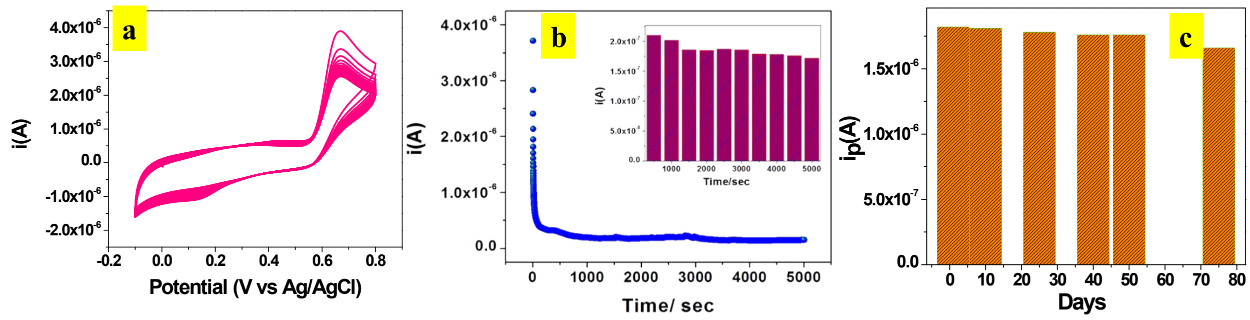

Fig. 2S. (a) Repeatability study using CV for 100 repetative measurements with 124.3 nM POM and (b) Amperometric stability study for the TiO_2-MoO_3-BMIMBr /GCE with 124.3 nM POM addition. Insert shows flow chart plot of amperometric current (500 s interval) vs time. (c) Flow chart of peak current vs days

References

1 A. Kutluay and M. Aslanoglu, *Sensors Actuators, B Chem.*, 2013, **185**, 398–404.
